# Teleworking Impact on Wellbeing and Productivity: A Cluster Analysis of the Romanian Graduate Employees

**DOI:** 10.3389/fpsyg.2022.856196

**Published:** 2022-02-25

**Authors:** Ştefan-Alexandru Catană, Sorin-George Toma, Cosmin Imbrişcă, Marin Burcea

**Affiliations:** ^1^Department of Business Administration, Faculty of Business and Administration, University of Bucharest, Bucharest, Romania; ^2^Department of Applied Economics and Quantitative Analysis, Faculty of Business and Administration, University of Bucharest, Bucharest, Romania; ^3^Department of Public Administration, Faculty of Business and Administration, University of Bucharest, Bucharest, Romania

**Keywords:** teleworking, wellbeing, productivity, cluster analysis, company, Romanian graduate employees

## Abstract

The COVID-19 pandemic has already had an enormous impact on numerous aspects of human society such as health, education, economy, business, or work and created favorable conditions for the expansion of teleworking. The aim of the paper is to identify and analyze five teleworking impact factors that affect thewellbeing and productivity of employees. The data were gathered by a quantitative research method through a questionnaire applied to 327 Romanian employees who hold a Bachelor or Master degree. Firstly, they were analyzed and interpreted through a factorial analysis focusing on the five teleworking impact factors. Secondly, the authors carried on cluster analysis, followed by multiple linear regression, using R statistical software. This study shows that there is a plethora of factors that influence the wellbeing and productivity of employees: individual and societal factors, organizational and work-related factors, technological factors, social factors at home, and social factors at work. Also, the cluster analysis brings to light significant differences between various Romanian employees such as: their gender, income, age, education, and city size.

## Introduction

Since March 2020, the COVID-19 pandemic has already had an enormous impact on numerous aspects of human society such as health, education, economy, business, or work. The spread of the coronavirus disease and the severe nationwide lockdowns have led to several changes in the way businesses are operating around the world. At the beginning of 2020, with the outbreak of coronavirus disease, many worldwide governments recommended that companies facilitate teleworking to avoid employees gathering together in the same place (Belzunegui-Eraso and Erro-Garcés, 2020). On March 16, 2020, Romanian authorities declared a state of emergency and asked companies to promote teleworking as a measure to protect their employees and to reduce the threats of coronavirus. Although the legal framework on telework was adopted in Romania in 2018 (iFlow, 2020), the implementation of teleworking was moved more slowly than expected. However, the crisis generated by the COVID-19 pandemic made it suddenly experience a rebound. On the one hand, the mass introduction of the rapid advances in information and communication technologies has brought productivity gains and cost reductions for business organizations (Mirchandani, 2000; Gregg, 2011; Kanellopoulos, 2011). On the other hand, the spread of teleworking on an impressive scale in various industries and domains (e.g., banking, education, insurance, software) has significantly influenced the organizational culture and work processes of companies and the behavior of their employees (Gálvez et al., 2020). Without necessarily meaning the end of the traditional way of work the new era of digital workplaces has already begun and will expand in the next years.

### The Teleworking Concept

The term “telework” was introduced in 1976 (Nilles et al., 1976). It was widely spread primarily at the beginning of the 21st century as a new form of labor organization that could provide solutions to many individual, social and organizational problems (Bajzikova et al., 2016; Gálvez et al., 2020). teleworking is seen as a form of flexible work arrangement that entails working remotely from an employee for a large proportion of the employer’s time (Thompson and Vivien, 1998). Other researchers define the concept as “working from home by deploying information and communication technologies to keep in touch with colleagues and deal with allocated working tasks” (Nguyen and Armoogum, 2021, p. 4). Employees are free to work outside the office, usually facilitated by virtual communication tools such as teleconferences, videoconferences and intranets with remote log-in. Moreover, they can also decide when they work, defining their work schedules with flexible start and end times (Coenen and Wok, 2014). In essence, the focused literature analyzed teleworking from a wide variety of perspectives as researchers have provided many definitions of the concept. These are based on several major themes, such as organization, location and technology (Martino and Wirth, 1990).

Among the major transformations that have occurred over the recent decades in the world of work, the rapid expansion of flexible working practices has proved to be a valuable solution for many companies and institutions (OECD, 1995, 2016; Matli, 2020). Flexible working has become a concept that captures a plethora of working arrangements and a mantra for promoting the idea of working everywhere and anytime. In other words, it is designed to address both employer and employee needs in a mutually advantageous manner. There are different modalities of flexible working practices, such as flexible locations, flexible time, and flexible contracts, or a combination among them. Consequently, the following five main types of teleworking are encountered in the world of work (Morgan, 2004; Hislop and Axtell, 2009):

- mobile telework—the worker is not located at any one site but travels in order to maximize the delivery of services or capabilities (e.g., between customer and employer premises);
- home-based telework—the worker carries out his/her work-based activities from home;
- telecentres—there are local facilities where people seek to reduce the burden and cost of commuting to a central location;
- functional relocation—business functions are concentrated and delivered from distance; and
- telecottages—there are facilities locally-based that offer the teleworking community the opportunity for personal interaction, skills development, and high-performance information and communication technologies.

Telework intensity differs according to the amount of telework time that ranges from part-time to full-time telework (Perez Perez et al., 2003; Gajendran and Harrison, 2007). Part-time telework happens when a teleworker works partly from home, partly from the office or from a client site, while full-time telework occurs when he/she works from home or a place other than an office using telecommunication technologies all the time (Nakrošiene et al., 2019).

Telework use is moderate in teams where members worked outside the office once or twice per week or during up to eight working hours—the approximate equivalent of one working day—and extensive when team members worked outside the office more than twice per week or more than 8 h per week (Coenen and Wok, 2014). Some studies found that computer-mediated communication, especially email, could effectively support knowledge sharing in teleworking teams that lack the ability to engage in face-to-face communication (Lee et al., 2007).

There is a plethora of factors that influence teleworking, such as: individual factors, job and organizational factors, family/home factors (Baruch and Nicholson, 1997), and socio-demographic characteristics (e.g., gender, age, number of children, and marital status; Nakrošiene et al., 2019). On the other hand, the job demands-resources theory considers that working conditions can be divided into job demands (e.g., physical workload, time pressure, recipient contract, physical environment, and shift work) and job resources (e.g., rewards, feedback, participation, job control, job security, and supervisor’s support), and evaluates the effects of different teleworking impact factors on work outcomes (Demerouti et al., 2001). Consequently, higher job demands lead to strain and health impairment, and higher resources generate better performances (Parker et al., 2017).

According to Nilles (1991), technological factors increase organizational effectiveness and productivity, maximize the utilization of resources, improve wellbeing and job satisfaction within teleworking, increase equipment costs and raise technical issues (Pérez et al., 2007). Moreover, Haddon and Brynin (2005) consider that specific technologies may define different types of teleworking, and other authors determined the relevance of home office or satellite office location to workers’ measurable productivity levels and efficiencies (Garret and Danziger, 2007).

Teleworking has received substantial attention in the scientific community, with regard to the impact of its arrangements on individual teleworkers, including their social relationships, work–family conflict, job satisfaction, organizational commitment, and job performance (Kossek et al., 2006; Gajendran and Harrison, 2007; Martin and MacDonnell, 2012; Richardson and McKenna, 2014; Biron and van Veldhoven, 2016; Stripe and Zarraga-Oberty, 2017; Groen et al., 2018). As a consequence, teleworking is a relatively controversial concept in the literature, due to both its strengths and weaknesses (Gálvez et al., 2020; Nguyen and Armoogum, 2021). There are three viewpoints from which the advantages and disadvantages are presented: individual, organizational, and societal (Harpaz, 2002). From the individual point of view, the main benefits of teleworking are the potential to blend various aspects of people’s professional and personal lives (Dima et al., 2019), a better work-life balance (Baruch, 2000; Zhang et al., 2020). However, other authors reveal the pernicious impact of TW, portraying teleworkers as being overworked (Vega et al., 2015; Sorensen, 2017). From the organizational perspective, the main advantages of teleworking are the expansion of productivity, the increasing presence of the employees at work, and the establishment of a positive organizational image (Ward and Shabha, 2001). Nevertheless, the existing researches showed that the more teleworkers work from home, the less possibility they have of gaining work support from their supervisors (Lapierre et al., 2015) and lower visibility in the company (Cooper and Kurkland, 2002). Moreover, other disadvantages of teleworking are the decreasing time for conversations with colleagues (Wilson and Greenhill, 2004), the transformation of work processes, and the legal issues (Harpaz, 2002). Teleworkers may feel a state of loneliness (Bailey and Kurkland, 2002), a lack of cooperation and communication with colleagues, and insufficient social interaction. That leads to the decrease of their organizational identification (Ammons and Markham, 2004). Thus, trust is a necessary condition for interpersonal cooperation, and it can be diminished when employees interact less frequently (McAllister, 1995). From the societal viewpoint, the main advantages of teleworking are the reduction of environmental damage, the decrease of traffic congestion, the provision of solutions for special-needs populations and the decrease of energy consumption, while the main disadvantages are the unclear legal issues and the creation of a detached society (Harpaz, 2002).

### The Impact of Teleworking on Wellbeing and Productivity

In the literature, there are several studies that present and analyze the relationships between teleworking and the wellbeing (Azarbouyeh and Naini, 2014; Miron et al., 2021) and productivity (Ward and Shabha, 2001) of employees. In the last decades, more and more employers have focused their attention on the wellbeing of their employees by providing a healthy, pleasant, and supportive workplace (Weinberg and Doyle, 2017), and various stimulative benefits (Harter et al., 2003). The increase of the employees’ wellbeing makes them more engaged and, therefore, leads them to obtain a higher productivity (Beheshti, 2019). As the employees gain higher outputs, they feel a “heightened sense of wellbeing” (Bosua et al., 2013, p. 11.9).

During the time, various points of view from several domains (e.g., psychology, medicine, and sociology) have been expressed related to the concept of wellbeing. Thus, wellbeing constitutes not only a broad, complex, multi-dimensional, and multifaceted concept (Catană et al., 2021) but also a state (Dodge et al., 2012). However, two different perspectives seem to be predominant as they operationalize wellbeing as follows:

- The so-called “clinical tradition” emphasizes the need to measure depression, distress, anxiety, or substance abuse (Thoits, 1992).
- The so-called “psychological tradition” highlights one’s subjective evaluation of life through satisfaction and affect or personal functioning (Keyes, 1998).

As a key human need, wellbeing is often associated and even considered synonym with concepts such as welfare, life satisfaction or quality of life, good health, autonomy, happiness, purpose in life, self-acceptance, comfort, prosperity, security, positive relationships with others or making contributions to the community (Ryff, 1989; Lindberg, 2002; Shah and Marks, 2004) and is deeply linked with a “good or satisfactory condition of existence” (Webster’s Encyclopedic Unabridged Dictionary of the English Language, 1996, p. 1620). It represents a “global assessment of a person’s quality of life according to his own chosen criteria” (Shin and Johnson, 1978, p. 478), a “balance point between an individual’s resource pool and the challenges faced” (Dodge et al., 2012, p. 230), and reflects “feelings about oneself in relation to the world” (Clements-Croome, 2005, p. 27). Several types of wellbeing have been found in the literature as follows:

- Subjective wellbeing is seen as a “person’s cognitive and affective evaluations of his or her life as a ‘whole’ (Diener et al., 2009, p. 187) and a rather fluctuating state (Headey and Wearing, 1991).” It comprises life satisfaction, pleasant and unpleasant effects (Diener and Suh, 1997).
- Psychological wellbeing is considered as the absence of both dysfunction and distress (Joseph and Wood, 2010) and embraces various affective aspects of daily experience (Warr, 1978).
- Social wellbeing represents “the appraisal of one’s circumstance and functioning in society” (Keyes, 1998, p.122) and encompasses social integration, social contribution, social coherence, social actualization, and social acceptance.

At the organizational level, employee wellbeing refers to his/her psychological and physical health (Edwards, 1992). Thus, it integrates both his/her psychological wellbeing (e.g., depression, anxiety) and physical wellbeing (e.g., heart rate, blood pressure). Researches emphasize that a higher employee wellbeing is considerably associated with “better job performance, lower absenteeism, reduced probability of leaving an employer, and the occurrence of more discretionary work behaviors” (Warr, 1999, p. 392).

On its turn, productivity is an economic indicator, that measures “the efficiency of production, taking the form of a ratio of the output of goods and services to the input of factors of production” (The Chartered Management Institute, 2004, p. 342). Consequently, employee productivity expresses the rate of output per employee during his/her working time. In other words, it measures the individual employee’s output in a given amount of time. However, the employees’ perceptions of their productivity level may differ in comparison to their real value of productivity. In this respect, researchers assert that an accurate measurement of the employee productivity during teleworking is rather difficult to achieve without a careful review of how managers and employees perceive it (Bosua et al., 2017). Employee productivity is positively influenced by technological factors (e.g., modern equipment, information communication technologies), psychological factors (e.g., encourage and praise), social factors (e.g., flexible work schedule, workplace conditions), individual factors (e.g., knowledge, abilities) and/or managerial factors (e.g., participative management, quality circles). Based on all these findings, the authors summarize some of the main effects of the teleworking impact on wellbeing and productivity (Table 1).

**Table 1:** Teleworking impact on wellbeing and productivity.

**Positive effects**	**Negative effects**
increased individual’s work-life balance (Ammons and Markham, 2004); harmonizing various facets of people’s lives (Dima et al., 2019); taking care of family members (Johnson et al., 2007); increased employees’ free time (Azarbouyeh and Naini, 2014); deeper integration between work and family roles (Raghuram and Wiesenfeld, 2004); time-planning autonomy (Gurstein, 2001; Morgan, 2004); increased individual’s flexibility and autonomy (Chapman et al., 1995); preserving employees’ energy (Azarbouyeh and Naini, 2014); increased productivity (Ward and Shabha, 2001); increased provision of human resources (Harpaz, 2002); savings in direct expenses (Ward and Shabha, 2001); creation of a positive organizational image (Harpaz, 2002); increased career opportunities for women (Schreiber, 1999); reduced temporal and spatial constraints in daily schedules (Pendyala et al., 1991); reduced stress (Sousa-Poza and Sousa-Poza, 2000); higher job satisfaction (Fonner and Roloff, 2010); enhanced job-related attitude (Gajendran and Harrison, 2007); increased value of the psychological contract employees has with their organization (Scandura and Lankau, 1997).	unbalanced work-life relations (Bailey and Kurkland, 2002); increased the permeability of work and family boundaries (Igbaria and Guimaraes, 1999). increased working time (Johnson et al., 2007); frequent work interruptions (Bailey and Kurkland, 2002); less support from others at work, especially from supervisors (Lapierre et al., 2015); lack of recognition from supervisors (Nohara et al., 2010); lower visibility of teleworkers (Cooper and Kurkland, 2002); reduced time for communication with colleagues (Wilson and Greenhill, 2004); frequent changes in work methods (Harpaz, 2002); new different legal issues (Harpaz, 2002); increased social isolation (Bailey and Kurkland, 2002); diminished social presence (Short et al., 1976); decreased the organizational identification of teleworkers (Cooper and Kurkland, 2002);

### Research Model, Objectives and Hypothesis

Starting from the above-mentioned considerations, two research objectives were established as follows:

Objective 1 (O1): To identify some of the main teleworking impact factors on wellbeing and productivity and to present their items.

Objective 2 (O2): To benchmark the effects of teleworking impact factors on wellbeing and productivity of Romanian employees grouped in three clusters.

**Figure 1:**
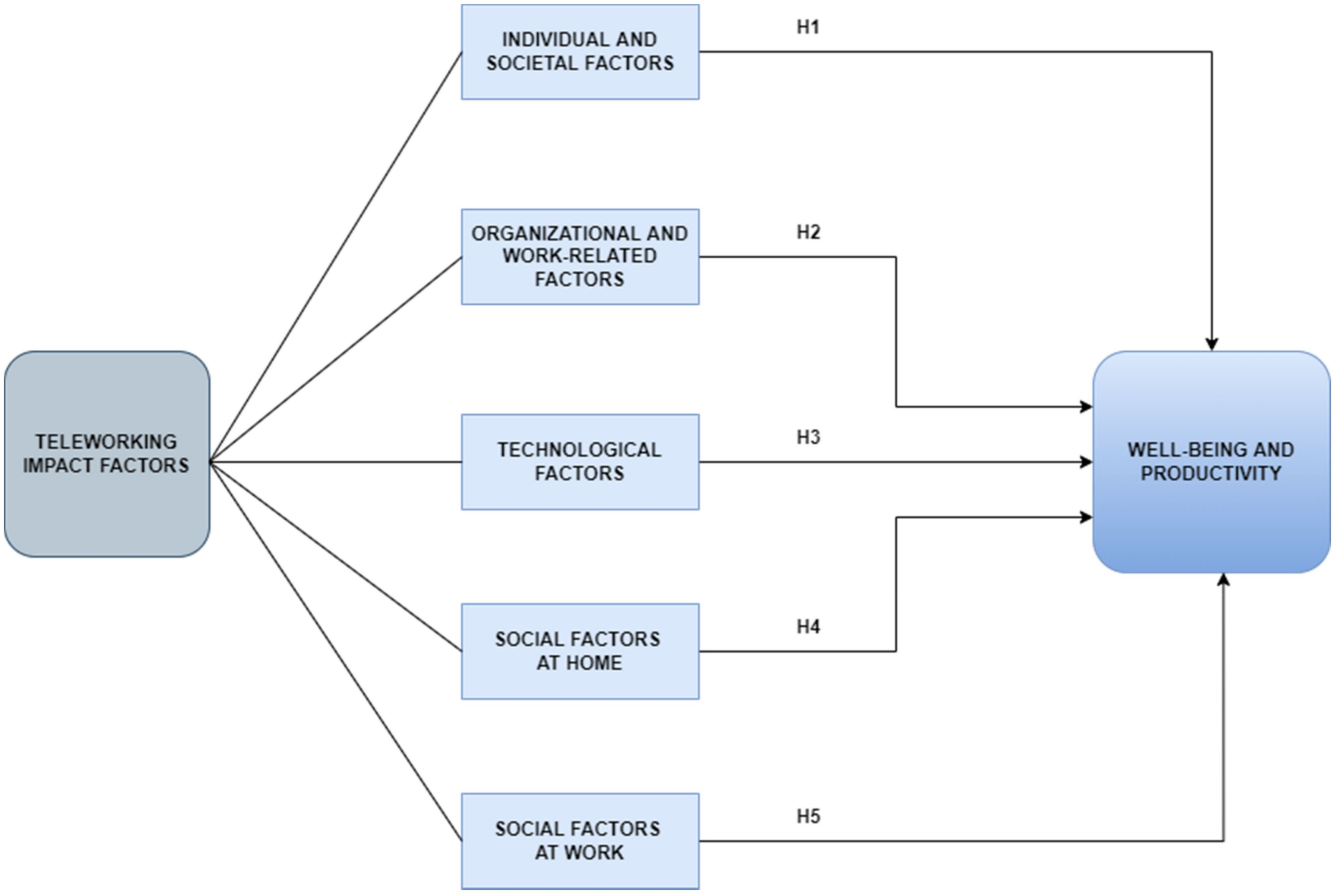
Research model.

The authors have designed and empirically tested the theoretical model to show the impact of five TW factors on the wellbeing and productivity of graduate employees (Figure 1): individual and societal factors, organizational and work-related factors, technological factors, social factors at home, and social factors at work. Each factor is defined through a different number of items. The dependent variables are the wellbeing and productivity of the employees during teleworking and the independent variables are the five teleworking factors.

Starting from the above objectives, the following five research hypotheses were set up:

*Hypothesis 1 (H1)*: Individual and societal factors positively influence the employees’ perceptions on their wellbeing and productivity.

*Hypothesis 2 (H2)*: Organizational and work-related factors positively influence the employees’ perceptions on their wellbeing and productivity.

*Hypothesis 3 (H3)*: Technological factors positively influence the employees’ perceptions on their wellbeing and productivity.

*Hypothesis 4 (H4)*: Social factors at home positively influence the employees’ perceptions on their wellbeing and productivity.

*Hypothesis 5 (H5)*: Social factors at work positively influence the employees’ perceptions on their wellbeing and productivity.

Against this background, this study aims to identify and analyze the above five teleworking impact factors that affect wellbeing and **productivity** of employees. To reach these objectives, the authors used a quantitative research method through a questionnaire applied to the Romanian employees who hold a Bachelor or Master degree.

The paper is structured as follows. Section 2 illustrates the materials and methods. Results and discussion are revealed in Sections 3, and 4, respectively. Section 5 displays the conclusions, along with their limitations and research perspectives.

## Materials and Methods

In order to reach the aims of the paper, the authors went through several phases of the research process. The first step was to search for information through desk research. A plethora of information (e.g., articles, books, and dictionaries) from the domains of psychology, economics and sociology were identified and collected from electronic databases (e.g., ScienceDirect) and libraries (e.g., the Central University Library Carol I of Bucharest). The literature review supported the authors in the design of the questionnaire.

### Questionnaire Design and Sample Selection

The authors designed and elaborated the questionnaire, after reviewing the literature. It contains 50 items, out of which seven refer to socio-demographic information of the employees (gender, age, education level, income, location, marital, and parental status), nine questions regarding the advantages of teleworking, 13 questions regarding teleworking impact factors (Table 2), and nine questions related to their employment situation (Table 3). The others were not used as they did not show significant results. The respondents’ perceptions were measured by using the Likert scale from 1 (minimum) to 5 (maximum).

**Table 2:** Testing data from employees’ perception.

**Items**	**Factor loadings**	**Factor**	**Cronbach’s *ɑ***
Time saved in traffic, by the employee	0.73	Individual and societal factors	0.88
Reduced pollution	0.63		
Possibility to work from home when the employee has a health issue	0.97		
People with disabilities could work	0.97		
A more careful society about the needs of the individuals	0.68		
Increased work productivity	0.77	Organizational and work-related factors	0.74
Cost reduction for employer	0.57		
Improved work-life balance	0.75		
A more flexible working schedule	0.59		
Slow internet speed	0.52	Technological factors	0.85
Lack of IT support from the company	0.56		
Limited access to technology	0.64		
Insufficient IT skills	0.92		
Lack of IT security solutions	0.83		
Involvement in household activities	0.91	Social factors at home	0.79
Care for children and the elderly	0.83		
Lack of adequate workspace	0.58		
Difficulty in separating work and household activities	0.62		
Reduced ability to focus	0.58		
Lack of social interactions with colleagues	0.89	Social factors at work	0.83
Difficulty in separating work and household activities	0.56		
Reduced ability to focus	0.52		
Difficulty in managing the relationship with clients and collaborators	0.57		
Social isolation	0.93		

**Table 3:** Employment and teleworking conditions per cluster.

		**Cluster 1**	**Cluster 2**	**Cluster 3**	**Total**
Company size	0–9 employees	22 (15.38%)	20 (16.39%)	8 (12.90%)	50 (15.29%)
	10–49 employees	31 (21.68%)	15 (12.29%)	12 (19.35%)	58 (17.73%)
	50–249 employees	48 (33.56%)	43 (35.24%)	19 (30.64%)	110 (33.63%)
	Over 250 employees	42 (29.37%)	44 (36.06%)	23 (37.09%)	109 (33.33%)
Type of position in the company^*^	Managerial	40 (27.97%)	48 (39.34%)	13 (20.96%)	101 (30.88%)
	Employee	103 (72.02%)	74 (60.65%)	49 (79.03%)	226 (69.11%)
Company allows teleworking	All the time	72 (50.34%)	77 (63.11%)	37 (59.77%)	186 (56.88%)
	Some of the time	71 (49.65%)	45 (36.88%)	25 (40.32%)	141 (43.11%)
					
The company allows a flexible working schedule	Yes	49 (34.27%)	34 (27.86%)	13 (20.96%)	96 (29.35%)
	No	94 (65.73%)	88 (72.13%)	49 (79.03%)	231 (70.64%)
The company^*^	Offers full access to teleworking technologies	83 (58.04%)	87 (71.31%)	33 (53.22%)	203 (62.08%)
	Has the necessary infrastructure, but it is rarely used	27 (18.88%)	20 (16.39%)	20 (32.25%)	67 (20.49%)
	Has the infrastructure, but does not use it	2 (1.39%)	3 (2.45%)	1 (1.61%)	6 (1.84%)
	Does not offer any technical support	31 (21.67%)	12 (9.83%)	8 (12.90%)	51 (15.59%)
Did your require IT support from your company, during teleworking?^*^	Yes	73 (51.04%)	35 (28.68%)	32 (51.61%)	140 (42.81%)
	No	70 (48.95%)	87 (71.31%)	30 (48.38%)	187 (57.18%)
Are you able to finish your tasks during the working schedule?^*^	Yes	69 (48.25%)	103 (84.42%)	42 (67.74%)	214 (65.44%)
	No	74 (51.73%)	19 (15.58%)	20 (32.25%)	113 (34.56%)
How do you evaluate your work P during teleworking in comparison with working from the office?^*^	Higher	42 (29.37%)	62 (50.81%)	24 (38.7%)	128 (39.14%)
	The same	93 (65.03%)	50 (40.98%)	31 (50%)	174 (53.21%)
	Smaller	8 (5.59%)	10 (8.19%)	7 (11.3%)	25 (7.65%)

* Existence of significant differences between the variables.

The numbers represent the respondents of each cluster, and the values in parentheses show the percentages per cluster.

The authors used a quantitative research method, based on a survey. The surveying technique was carried on through the Computer-assisted web interviewing (CAWI). Some authors consider CAWI to be synonymous with conducting a web survey (Biffignandi and Bethlehem, 2021, p. 68) whereas others consider that the notion is not correct because “interviewing” implies the presence or involvement of an interviewer, which is not the case for web surveys (Callegaro et al., 2015, p. 51). The stratified sampling was used as a sampling method. It represents a data-gathering method in which participants are chosen based on predetermined criteria (employees with bachelor/master degree) so that the final sample has the same characteristics as the studied population (Taherdoost, 2016). The study was focused on Romanian people with at least graduade level of education, with a focus on the latter, who are employed in the areas of activity mentioned in Table 4. The information that are available from the Romanian National Institute of Statistics (2021a) refer to the types of occupation (Romanian National Institute of Statistics, 2021b; Romanian National Institute of Statistics, 2021c) specific to various fields of activity (CAEN, 2022).

**Table 4:** Distribution of respondents by area of activity and level of education.

**Area of activity**	**Bachelor degree (%)**	**Master degree (%)**	**Total (%)**
Public administration	13.0	18.2	15.9
Commerce/sales/business consultancy	21.2	34.3	28.4
Education, research or communication	9.6	13.3	11.6
Finance, banking or insurance	17.8	3.9	10.1
IT	4.1	7.7	6.1
Medical	23.3	13.3	17.7
Non-financial services	11.0	9.4	10.1
Total	100.0	100.0	100.0
Number or participants (*n*)	146	181	327

The questionnaires were distributed by using the Google Forms platform, preserving the anonymity of the respondents. The data were collected between the 18th of January 2021 and the 17th of February 2021, through CAWI. After receiving, centralizing, and systematizing the answers gathered online, 327 questionnaires were validated from 373 responses (87.6%). The average completion time was 8 min. The questionnaire was applied to employees working in areas that are suitable for teleworking (e.g., education, public administration) especially due to the COVID-19 pandemic. Authors applied inclusion/exclusion criteria. The questionnaire was addressed only to teleworking graduate employees (Table 4).

### Data Analysis

The collected data were analyzed through the factor analysis that puts together “common variables into descriptive categories” (Yong and Pearce, 2013, p. 80). After identifying the effects of the five teleworking impact factors, the authors carried on cluster analysis in order to see how the respondents can be segmented and what are their defining characteristics. The cluster analysis aims to create homogeneous groups by assigning observations to bunches of people, so that these being similar, with respect to specific attributes of interest (Tryfos, 2005). Moreover, the authors handled a multiple linear regression in order to determine the way these factors influence the wellbeing and productivity of the employees. The analysis was conducted in R (version 4.0.5) using the RStudio (version 1.4.1106) user interface. The packages used in this analysis were tidyverse (1.3.0), psych (2.0.9), nFactors (2.4.1), psycho (0.5.0), psy (1.1), clustertend (1.5), factoextra (1.0.7), NbClust (3.0), and dendextend (1.14.0). A screeplot with the Eigen values and parallel analysis were performed. A factorial analysis was done in order to identify the factor loadings of the variables from the questionnaire. Promax rotation was used in order to maximize variance and to minimize items loading on all five factors (Table 2).

Cronbach’s *ɑ* was used to measure internal consistency of the questionnaire and the values were 0.88 for individual and societal factors, 0.74 for organizational and work-related factors, 0.85 for technological factors, 0.79 for social factors at home, and 0.83 for social factors at work. All the values are above 0.7 and dropping any of the items would lead to a lower value (Drost, 2011). The maximum likelihood method was used to extract the factors (Heeler et al., 1977).

The authors chose the cluster analysis in order to better analyze the data. Consequently, K-means clustering was preferred as it is one of the most popular clustering methods used (Towards data science, 2018). The authors computed Hopkins’ statistic, using the R package factoextra (The Comprehensive R Archive Network, 2021), which was determined to be 0.68, greater than 0.5 which showed there existed a clustering tendency among the 5 identified factors (Lawson and Jurs, 1990).

The authors used the package NbClust (Charrad et al., 2014) in order to identify the optimal number of clusters. By using various indexes and methods, including the popular average silhouette method, the elbow method, and the Euclidean distance method, the proper conclusion was the use of 3 clusters. The clusters descriptions are presented in the following section of the paper. Statistically significant differences are identified using Kruskal–Wallis analysis as the results do not respect the homogeneity or normality requirements of ANOVA test. The three clusters were benchmarked by taking into account the five teleworking impact factors, the socio-demographic and economic descriptors, and the employment teleworking conditions.

## Results

### Clusters’ Identification

The authors identified three clusters of employees, by taking into account their specific homogeneity. In this respect, they focused on the values of the variables used in the cluster identification process (Table 5). The values are shown for the entire dataset as well as per cluster. Furthermore, the authors tested for statistically significant differences between clusters using Kruskal–Wallis one-way analysis of variance for variables that had ordered values and the Chi-squared test for nominal variables. The analysis of the results led to the identification of three clusters (Table 5).

**Table 5:** Factor average scores per cluster.

	**Cluster 1**	**Cluster 2**	**Cluster 3**
Individual and societal factors^*^	0.18	0.73	−1.45
Organizational and work-related factors^*^	−0.05	0.58	−1.04
Technological factors^*^	0.71^*^	−0.61	−0.44
Social factors at home^*^	0.77	−0.68	−0.43
Social factors at work^*^	0.77	−0.70	−0.40

* Existence of significant differences between the variables.

The cluster differences were tested using Kruskal–Wallis analysis. All these differences were significant at the 95% level with the exception for clusters 2 and 3 with regard to technological factors.

Based on the above data, the results of our analysis show that clusters 2 and 3 are fairly similar with regard to the last 3 teleworking impact factors identified in the previous analysis whereas cluster 1 stands out quite a bite.

Technological factors, social factors at home and at work are of less concern for both clusters 2 and 3. The employees from cluster 2 stand out due to the fact that they consider that work from home is beneficial as it allows increased work productivity, more flexible schedule but also certain individual and societal gains like reduced pollution, and inclusiveness of people with disabilities. Individual and societal factors, and organizational and work-related factors are of very little importance for the employees of cluster 3.

Cluster 1 is markedly different in terms of the last three teleworking impact factors. The employees experienced significant technological issues, such as: insufficient IT skills, involvement in household activities, social isolation. Both individual and societal factors and organizational and work-related factors are of little importance.

### Socio-Demographic and Economic Descriptors Per Cluster

The authors presented the socio-demographic and economic descriptors of the employees from each cluster (Table 6). The average age of all the respondents was 39.7 years old, with the following differences between the clusters: 39.65 for cluster 1, 38.71 for cluster 2, and 42.75 for cluster 3.

**Table 6:** Socio-demographic and economic descriptors per cluster.

		**Cluster 1**	**Cluster 2**	**Cluster 3**	**Total**
Gender	Male	42 (29.37%)	46 (37.70%)	24 (38.70%)	112 (34.25%)
	Female	101 (70.63%)	76 (62.29%)	38 (61.29%)	215 (65.75%)
Income ($1 = 4.03 RON)	<1.500 RON (<$350)	3 (2.09%)	0 (0%)	0 (0%)	3 (0.91%)
	1.500–3.000 ($351–$750)	25 (17.48%)	13 (10.65%)	7 (11.29%)	45 (13.76%)
	3.001–4.500 ($751–$1.100)	48 (33.56%)	29 (23.77%)	18 (29.03%)	95 (29.51%)
	4.501–6.000 ($1.101–$1.500)	36 (25.17%)	30 (24.59%)	16 (25.80%)	82 (25.07%)
	>6.000 RON (>$1.500)	31 (21.67%)	50 (40.98%)	21 (33.87%)	102 (31.19%)
					
Education	Bachelor degree	59 (41.25%)	57 (46.72%)	30 (48.38%)	146 (44.64%)
	Master degree	84 (58.74%)	65 (53.27%)	32 (51.61%)	181 (55.35%)
City size	Rural	9 (6.29%)	8 (6.55%)	3 (4.83%)	20 (6.11%)
	<30.000	3 (2.09%)	8 (6.55%)	2 (3.22%)	13 (3.97%)
	30.000–100.000	7 (4.89%)	4 (3.27%)	3 (4.83%)	14 (4.28%)
	100.001–200.000	6 (4.19%)	9 (7.37%)	5 (8.06%)	20 (6.11%)
	>200.000	118 (82.51%)	93 (76.22%)	49 (79.03%)	260 (79.51%)
Marital status	Not married	48 (33.56%)	44 (36.16%)	20 (32.25%)	112 (34.25%)
	Married	73 (51.04%)	57 (46.72%)	35 (56.45%)	165 (50.45%)
	Cohabitation	9 (6.29%)	5 (4.09%)	3 (4.83%)	17 (5.19%)
	Divorced	13 (9.09%)	15 (12.29%)	3 (4.83%)	31 (9.48%)
	Widower	0 (0%)	1 (0.81%)	1 (1.61%)	2 (0.61%)
Children	Yes	70 (48.96%)	58 (47.54%)	20 (32.25%)	148 (45.25%)
	No	73 (54.04%)	64 (54.45%)	42 (67.75%)	179 (54.75%)

The numbers represent the respondents of each cluster, and the values in parentheses show the percentages per cluster.

The analysis of these outcomes highlights the following:

- By taking into account the gender of employees, females are more predominant in cluster 1 in comparison with clusters 2 and 3.
- By considering the income, more than half of the employees from cluster 1 have an income below $1.100 whereas the majority of employees from clusters 2 and 3 have an income above $1.100.
- By taking into account the medium value of the age of respondents, the employees from clusters 1 and 2 are below 40 years old in comparison with the employees from cluster 3, which are above 42 years old.
- In terms of education, the employees from cluster 1 are more educated than the employees from clusters 2 and 3.
- By taking into account the city size, the majority of employees from cluster 1 are located in big cities in contrast with the employees from clusters 2 and 3.
- In terms of their marital status, more than half of the employees from clusters 1 and 3 are married whereas the majority of the employees from cluster 2 are not married.
- By considering their children, the proportion of employees with children is much higher in clusters 1 and 2 in comparison with cluster 3.

It is worth noting that the authors used the Kruskal-Wallis analysis to check the significant differences between the various groups and they found almost no differences. Income was the only one where it was found a statistical difference (Chi-square = 14.014, df = 2 and *p* = 0.0009) and, therefore, the Pairwise Wilcoxon Rank Sum tests was used in order to identify the differences. The only statistically significant difference, at the 95% CI, is between cluster 1 (the one with the lowest incomes), and cluster 2 (the one with the highest incomes). The authors consider that this result is not surprising, because people with different income levels will have different perceptions on wellbeing and productivity during teleworking.

### Employment and Teleworking Conditions Per Cluster

Moreover, the authors take into consideration the employment situation and teleworking conditions of the respondents (Table 3). The authors included a question related to COVID-19 pandemic and to what extent the employees consider that the decision of their employer to facilitate teleworking was influenced by the pandemic. The results are not presented because they are the same across all three clusters: the employees state that it had a significant impact (the average value was 4.48, on a Likert scale from 1—not important to 5—very important).

The results of our analysis demonstrated the following:

- The proportion of employees working in big companies is higher in clusters 2 and 3.
- Around 40% of the employees from cluster 2 occupies managerial positions whereas the vast majority of employees from clusters 1 and 3 hold executive positions.
- The companies from cluster 2 provide the best opportunities to telework all the time.
- The COVID-19 pandemic influenced in the same manner the companies’ decisions to adopt teleworking.
- The companies from clusters 1 and 2 provide a more flexible working schedule than the companies from cluster 3.
- More than 70% of the companies from cluster 2 provide full access to teleworking technologies whereas a little more than a half of the companies from clusters 1 and 3 do the same.
- The majority of employees from clusters 1 and 3 enjoyed full access to information and communication technologies and IT support in contrast with the employees from cluster 2.
- The vast majority of the employees from cluster 2 were able to accomplish their tasks during the working schedule, followed by the employees from cluster 3. Less than half of the employees of cluster 1 attained their assignments in time.
- The productivity of employees from cluster 2 is much higher than that of the employees from clusters 1 and 3.

## Discussion

Based on the factorial analysis, the outcomes of our research showed some of the positive and negative effects of teleworking impact factors on wellbeing and productivity. The authors identified and analyzed five teleworking impact factors: individual and societal factors, organizational and work-related factors, technological factors, social factors at home, and social factors at work. Accordingly, while previous studies described many items related to these factors (Ipsen et al., 2021), our research customized these results in the case of Romanian graduate employees.

The authors identified three clusters that are different in terms of the influence of each teleworking factor by taking into account socio-demographic characteristics, economic descriptors, and employment and teleworking conditions. The employees’ perceptions on their productivity during teleworking was measured by using two questions. The first was “Are you able to finish your tasks during the working schedule?” The authors checked for statistically significant differences using Kruskal–Wallis analysis (Chi-squared = 37.681, df = 2, value of *p* = 6.572e-09) and found significant differences between all three clusters. Then, the second question was “How do you evaluate your work productivity during teleworking in comparison with working from the office?” The authors tested the differences between the clusters with the same Kruskal–Wallis analysis (Chi-squared = 4.509, df = 2, value of *p* = 0.000707) and discovered no significant differences between clusters 2 and 3, but with some differences between them and cluster 1. Thus, cluster 1 is composed by employees who considered that they are more productive. They also needed the highest support from their company, but received the least. The employees from cluster 2 obtained the best results in terms of perceived productivity, being able to finish their work on time and receiving the most support from their company. They are also the most likely ones to hold a managerial position. Cluster 3 seems to be between clusters 1 and 2. The employees perceived an improved productivity, being able to finish work on time and receiving only some support from their employers. In essence, employees’ perceptions about the increase of their productivity was favorable.

The first research hypotheses (H1) states that individual and societal factors positively influence the employees’ perceptions on their wellbeing and productivity. The employees from cluster 2 consider these factors as important, in comparison with the employees from cluster 1 who were neutral and from cluster 3 who perceive them as the least important (Kruskal–Wallis, Chi-squared = 143.72, df = 2, *p* = 2.2 × 10^−16^). This means that teleworking is important for employees who are interested to save time in traffic, to be involved in societal issues, and want to reduce pollution. They value these aspects, have an increased level of wellbeing, and obtain better results at work. These outcomes are congruent with previous researches (Kowalski and Swanson, 2005; Raiborn and Butler, 2009; Catană et al., 2021).

Interesting enough is the fact that the results obtained regarding the second hypothesis (H2) are similar. The employees from cluster 2 state that organizational and work-related factors positively influence their perceptions on their wellbeing and productivity, whereas the employees from cluster 1 are neutral, and those from cluster 3 assert that these are not important (Kruskal–Wallis, Chi-squared = 136.95, df = 2, *p* = 2.2 × 10^−16^). The employees who value cost reduction for employer, achieve a better work-life balance and enjoy a more flexible work schedule. By better managing teleworking, they obtain higher work outcomes. Our results are in agreement with those of Apgar (1998), and Feng and Savani (2020).

Technological factors (H3) prove to be an important factor in positively influencing their perceptions on wellbeing and productivity. By using Kruskal–Wallis analysis, the results show that the employees from cluster 1 consider that these factors are not important (inverted questions in the questionnaire, a higher value means more problems with IT support, slow internet or limited skills.) whereas they are important for the employees from clusters 2 and 3 (Chi-Squared = 152.65, df = 2, *p* = 2.2 × 10^−16^). They perceive that technological factors play a paramount role. Also, the employees that encountered the least problems with technology are those who obtained the best results in terms of perceived productivity improvements. Our outcomes are similar to those obtained by Marzban et al. (2021), and Urbaniec et al. (2022).

Social factors at home (H4) have also a positively influence. There are significant differences among the three clusters (Kruskal–Wallis, Chi-squared = 175.36, df = 2, *p* = 2.2 × 10^−16^). Higher values mean that the employees encountered more problems regarding the lack of space, separating work and household activities, and their reduced ability to focus on work tasks. The highest values belong to the employees from cluster 1, which had the lowest results in terms of perceived productivity and wellbeing. The employees from cluster 2 and 3 scored the lowest values that means that improved conditions at home might have a significant impact on their performances. Other researches confirm our results (Allen et al., 2015; Feldman and Mazmanian, 2020).

Social factors at work (H5) prove to have a positively influence on the employees’ perceptions on their wellbeing and productivity. The employees from clusters 2 and 3 obtained the lowest (best) values in comparison with those from cluster 1 who score the highest (worst) values. The differences prove to be statistically significant (Kruskal–Wallis, Chi-squared = 136.95, df = 2, *p* = 2.2 × 10^−16^). The employees who encountered problems with social isolation, and have difficulties in getting in touch with colleagues, clients or collaborators, perceive that their performances drop. These results are congruent with those obtained by Raiborn and Butler (2009), Windeler et al., (2017), and Van der Voordt and Jensen (2021).

## Conclusion

The spread of the COVID-19 pandemic has significantly changed the way work processes are carried out. The originality of this research is three folds. Firstly, it consists in conducting an empirical study among the Romanian graduate employees, grouped in three clusters starting from their homogeneity. Secondly, this study identifies the five teleworking impact factors and design a specific research model that influence them to wellbeing and productivity of the employees. Thirdly, it analyzes the way in which these factors affect employees’ wellbeing and productivity, by benchmarking the three clusters.

The results of this research show that the following five teleworking impact factors influence employees’ perceptions on wellbeing and productivity: individual and societal factors, organizational and work-related factors, technological factors, social factors at home, and social factors at work. Each of them encompasses various items such as: possibility to work from home when the employee has a health issue, improved work-life balance, IT skills, involvement in household activities, and social isolation. The impact of these teleworking factors varies among the three identified clusters.

According to their Cronbach’s *ɑ* values, individual and societal factors, technological factors, and social factors at work represent the factors with the highest impact on the employees’ perceptions about their wellbeing and productivity (all of them above 0.83), followed by social factors at home, and organizational and work-related factors (both above 0.8).

The outcomes of this study demonstrate that these teleworking impact factors positively influence the employees’ perceptions on their wellbeing and productivity. However, there are some differences among the three analyzed clusters. In this respect, the employees from cluster 2 (income above $1.100, medium value of age below 40 years old, majority of them are not married, employed in big companies, 40% of them in managerial positions) consider that the influence of individual and societal factors, organizational and work-related factors, technological factors, and social factors at work is important. The employees from cluster 3 (income above $1.100, medium value of age above 42 years old, majority of them are married, employed in big companies, majority in executive positions) assert that technological factors, and social factors at work positively influence their perceptions on wellbeing and productivity. The perceptions of the employees from cluster 1 (income below $1.100, medium value of age below 40 years old, majority of them are married, employed in small and medium companies, majority in executive positions) are neutral in relation with the influence of individual and societal factors, organizational and work-related factors, and technological factors. All in all, employees’ perceptions about the enhancement of their wellbeing and the increase of their productivity were favorable, but at different rates among them.

From a theoretical point of view, this research brings to light the Romanian employees’ perceptions about teleworking impact on their wellbeing and productivity. Also, it contributes to the enrichment of the scientific literature on this topic. In addition, the paper provides a possible theoretical model that may clarify this subject.

From a practical point of view, teleworking should be implemented within the companies, by considering both their business purposes and the wishes and expectations of their employees. On the one hand, companies are interested in increasing their performances and, therefore, raising their employee productivity through teleworking. On the other hand, their employees are keen on their wellbeing enhancement. The obtained results are very important from a managerial perspective because they show that teleworking is a key aspect of the modern world and a vast majority of companies should make the transition to a working environment where teleworking is of a regular occurrence. Consequently, the companies may provide the proper technological infrastructure for their employees in order to enhance their wellbeing and increase their productivity. In order to be productive, when teleworking, the employees should have both technical support from their organizations and social support from their supervisors. If the company ignore these issues, and treats teleworking like a solution that works on its own, the results may turn out to be lackluster. Also, the companies can improve the work-life balance of their employees through the expansion of teleworking. Last but not least, the government may sustain and popularize a culture of teleworking through investment in modern technologies that may lead to increased employees’ wellbeing and productivity.

Concerning future research directions, other studies may take into account a larger number of teleworking factors and analyze their impact on employees’ perceptions on wellbeing and productivity. Moreover, they can reveal other items that can be correlated. Other authors might expand the research on other countries and geographical regions. Another limitation of this study is the size and the structure of the clusters, as it refers only to graduate Romanian employees. It is an imperfect measure, but it is the best one available before more data will be obtained in the future national census. Moreover, other socio-demographic, economic descriptors, and employment and teleworking conditions might be considered in other studies. A larger and more representative sample should be analyzed in future researches. Also, the actual representativeness of the sample is difficult to determine given the available data from the Romanian National Institute of Statistics. However, the national census that has started this year (2022) will provide valuable data that could be used for future researches.

## Data Availability Statement

The raw data supporting the conclusions of this article will be made available by the authors, without undue reservation.

## Ethics Statement

The study was conducted in accordance with the Declaration of Helsinki, and the protocol was approved by the Ethics Committee of the University of Bucharest (decision No. 14/25 February 2020). The patients/participants provided their written informed consent to participate in this study.

## Author Contributions

Ș-AC, S-GT, and MB contributed to the conception and design of the study. CI organized the database and performed the statistical analysis. Ș-AC and S-GT wrote the first draft of the manuscript. Ș-AC, S-GT, CI, and MB wrote sections of the manuscript. All authors contributed to manuscript revision, read, and approved the submitted version.

## Funding

The APC was funded by University of Bucharest.

## Conflict of Interest

The authors declare that the research was conducted in the absence of any commercial or financial relationships that could be construed as a potential conflict of interest.

## Publisher’s Note

All claims expressed in this article are solely those of the authors and do not necessarily represent those of their affiliated organizations, or those of the publisher, the editors and the reviewers. Any product that may be evaluated in this article, or claim that may be made by its manufacturer, is not guaranteed or endorsed by the publisher.
